# Contact Toxicity, Electrophysiology, Anti-Mating, and Repellent Effects of *Piper guineense* Against *Spodoptera frugiperda* (Lepidoptera: Noctuidae)

**DOI:** 10.3390/insects16090908

**Published:** 2025-09-01

**Authors:** Mobolade D. Akinbuluma, Jacques A. Deere, Peter Roessingh, Astrid T. Groot

**Affiliations:** 1 Department of Evolutionary and Population Biology, Institute for Biodiversity and Ecosystem Dynamics, 1098 XH Amsterdam, The Netherlands; j.a.deere@uva.nl (J.A.D.); p.roessingh@uva.nl (P.R.); a.t.groot@uva.nl (A.T.G.); 2 Department of Crop Protection and Environmental Biology, University of Ibadan, Ibadan 200005, Nigeria; 3 Faculty of Health and Medical Sciences, School of Biosciences, University of Surrey, Guildford, Surrey GU2 7XH, UK

**Keywords:** plant extract, lethal concentration, fall armyworm larvae, wind tunnel experiment, electroantennography

## Abstract

The fall armyworm, *Spodoptera frugiperda*, is a long-distance migratory pest, which invaded the African continent in 2016, causing enormous losses to agricultural crops, especially maize. Synthetic insecticides are primarily used for managing *S. frugiperda*, but they leave residues on human food and animal feed and also cause environmental hazards. We evaluated the crude ethanolic extract of *Piper guineense* fruits for contact toxicity on *S. frugiperda* larvae and determined the lethal concentration (LC_50_) of the extract. Additionally, we conducted an electrophysiological (EAG) experiment to determine the responses of adult *S. frugiperda* males and females to *P. guineense* and determined whether the extract influenced mating, oviposition, and repellence to the adult female. We found that *P. guineense* extract caused significantly higher mortality to *S. frugiperda* larvae than an ethanol control. Electrophysiologically, we observed significantly higher responses to the extract than the control, with some variations in response between the sexes. A wind tunnel experiment revealed that females moved more towards the control than towards the extract. Taken together, our results confirm that *P. guineense* extract is effective against *S. frugiperda* larvae and adults. Future research should explore the responses of *S. frugiperda* to *P. guineense* extract on a field scale.

## Simple Summary

The fall armyworm, *Spodoptera frugiperda* (Lepidoptera: Noctuidae), is a globally invasive, polyphagous pest that has established itself across all (sub)tropical agroecological zones. We studied the effect of *Piper guineense* extract on *Spodoptera frugiperda* larvae and adults. We found a positive relationship between *P. guineense* extract concentration and *S. frugiperda larval mortality.* We observed a sex difference in the antennal response of adult *S. frugiperda* to *P. guineense* extract. Additionally, we observed that *P. guineense* elicited a repellent effect on adult *S. frugiperda* females at close distance. Our findings highlight the potential application of *P. guineense* extract for control of *S. frugiperda.*

## 1. Introduction

The fall armyworm (*Spodoptera frugiperda* J.E. Smith) is a long-distance migratory pest, with adult moths capable of flying over 100 km in a single night [1]. The moth invaded the African continent in 2016 [2] and is now found throughout sub-Saharan Africa [3], Asia [4,5], and Australia [6]. It causes significant damage to many important crops like grass species and cereals, especially maize [7]. As maize is a primary staple food crop in Africa, the recent invasion of *S. frugiperda* threatens the food security of millions of people.

In the management of *S. frugiperda*, synthetic insecticides belonging to the organophosphates, carbamates, and pyrethroids are primarily used by farmers in Africa [8,9]. To mitigate the impact of *S. frugiperda* in Africa, governments have subsidized the use of synthetic insecticides as emergency control programs [10,11]. However, indiscriminate use of these chemicals has led to accumulation of chemical residues in food [12,13]. In addition, *S. frugiperda* is highly adaptable and well known to evolve resistance against synthetic pesticides [14,15], and this threatens the sustained use of pesticides.

A sustainable and efficient management of *S. frugiperda* should include an approach that is eco-friendly, without further harm to the environment. Incidentally, the use of plant parts and their extracts has been investigated as one of many potential low-cost control options that build on local knowledge and ecological principles [16,17]. In addition, since crop production is mostly done by many smallholders who lack financial resources to purchase the chemical pesticides [18], there is a need to explore more biodegradable and cheaper products to control *S. frugiperda.*

Some plant-based insecticides have already been found to cause mortality in *S. frugiperda* [19,20,21,22,23,24], and several of these plants have been recommended for field trials and bioassays [25,26]. Generally, plant extracts contain specific phytochemicals such as alkaloids, flavonoids, saponins, terpenoids, phenolic compounds, and quinones, which have the potential of showing acute toxicity, repellency, antifeedant, and growth regulation against many agricultural pests [27,28]. *Piper* species are among the plants whose insecticidal properties have been investigated as preventative against many insect pests, including *S. frugiperda* [24,29,30,31,32,33,34]. Application of extracts of *P. el-bancoanum* and *P. arboreum* at different dosages resulted in over 80% mortality rate of *S. frugiperda* larvae, similar to the application of *Bacillus thuringiensis* and the synthetic chemical chlorpyrifos [31].

In this study, we examined the extract of *P. guineense* (West African black pepper) due to its unique phytochemical profile [35] and its regional ecological relevance in sub-Saharan Africa [36,37]. Notably, *P. guineense* is rich in alkaloids, such as piperine, guineensine, piperlonguminine, trichostachine [38,39], and essential oils, including dillapiol, elemicine, myristicine, safrole [30,40], which contribute to its preservative and antioxidant properties [41]. Owing to its numerous constituents, extracts from *P. guineense* have demonstrated neurotoxic, oviposition-deterrent, growth-regulatory, and cytotoxic effects on multiple insect taxa [38,39,42,43,44]. However, to date, the effects of *P. guineense* on the full life cycle of *S. frugiperda*, particularly adult behavior, remain poorly understood. Understanding how plant-derived compounds affect adult behaviors remains a significant knowledge gap, yet this is essential for predicting and managing pest population dynamics.

Given its documented efficacy against lepidopteran pests in closely related species [45,46,47], we evaluated the effects of crude extract of *P. guineense* fruits not only on larvae but also on mating and oviposition behavior, as well as its potential repellent effect on adult female *S. frugiperda.* Additionally, we conducted electrophysiological experiments to determine antennal responses of male and female *S. frugiperda* adults when exposed to *P. guineense* extracts.

## 2. Materials and Methods

### 2.1. Insects

Populations of *Spodoptera frugiperda* from Kenya and Nigeria, hereinafter referred to as SFK and SFN, respectively, were used in this study. The SFK was collected from the lab population of the International Centre of Insect Physiology and Ecology (ICIPE), Kenya. The lab population was established with specimens collected from several areas in Kenya at different time points. At ICIPE, the population was kept at a temperature of 25 ± 2 °C, 70% ± 5% relative humidity, and a 12:12 h (light: darkness) photoperiod and on an artificial pinto bean diet. Samples of this laboratory population were received at the University of Amsterdam (UvA) in December 2020 and August 2022. The SFN was collected from maize fields in Southern Oyo State and from the International Institute of Tropical Agriculture (IITA), Ibadan, Nigeria, between January 2022 and May 2022 and was received at UvA in October 2022. Therefore, SFK was maintained under laboratory conditions at UvA for about 7–27 generations, while SFN had undergone approximately 5 generations before the start of the experiments in March 2023. At UvA, immature stages (eggs and larvae) of both populations were reared on an artificial pinto bean diet in climate chambers set at 25 °C and humidity of 60–65%, with a reversed light/dark cycle (lights off at 12.00, on at 22.00) and a 14:10 light/dark photoperiod. Adults were continuously fed with 10% sugar water, ad libitum.

### 2.2. Insecticidal Material and Preparation of Extract Concentrations

Fruit extract of *Piper guineense* Schum and Thonn (family Piperaceae) was used in this study. Fresh fruits of the plant were purchased from a local market in Kajola, Osun State, Nigeria (latitude 7°31′0.01″ and longitude 4°30′0″; terrain elevation of 257 m above sea level) in August 2021. Five hundred grams (500 g) of the fruits were air-dried and ground. The sample was extracted for 8 h using 2.5 L of 95% ethanol (Sigma–Aldrich, St. Louis, MO, USA) in a Soxhlet apparatus (Witeg Labortechnik GmbH, Wertheim, Germany) and was concentrated *in vacuo* on a rotary evaporator (Witeg Labortechnik GmbH, Wertheim, Germany) at 40 °C, under reduced pressure (150 mbar). A total of about two hundred grams (200 g) of the crude extract was obtained, yielding approximately 40% (*w*/*w*) relative to the starting plant material. The concentrated extract was stored in airtight amber glass bottles at −20 °C until use.

The sample was transferred to UvA in October 2022 and was further stored at −20 °C until the time of bioassays. To produce varying concentrations of the extract, 30 mL of the crude extract was dissolved in 70 mL of ethanol (95%) to a concentration of 30% (*v*/*v*) stock solution. The solution was homogenous, and no surfactant was needed, as the extract was completely soluble in ethanol. The stock solution was further diluted into concentrations of 15%, 3%, 1.5%, and 0.3% (*v*/*v*). These concentrations, including the stock, were used for bioassays against *S. frugiperda*, while ethanol only, i.e., 0% (*v*/*v*), was used as a negative control. All dilutions were freshly prepared immediately before bioassays.

### 2.3. Effects of Extracts on Larvae and Larval Development

To assess the toxicity of *P. guineense* extract on *S. frugiperda* larvae, third instar larvae were individually placed in a small cup (4 cm × 3 cm; 40 mL) and fed on an artificial pinto bean diet. Contact toxicity was performed by applying aliquots (10 μL) of each concentration of *P. guineense* extract to each larva using a micropipette (Eppendorf SE, Hamburg, Germany). A single application of the appropriate concentration was made dorsally on the thoracic region of each larva [28]. The cups were then covered with a lid. Each concentration was used to treat 20 *S. frugiperda* larvae of each population (SFN and SFK). Treated larvae were maintained under controlled laboratory conditions. Larval mortality was recorded one day, two days, and seven days post-treatment by counting the number of dead larvae. For each day of measurement, the median lethal concentration (LC_50_) of the extract concentration was determined. The larvae were considered dead if they displayed no observable response to a mechanical stimulus, i.e., short-term pressure applied with a camel hairbrush. Surviving larvae in each vial were followed through pupation. Emerging pupae were checked for survival and weighed daily.

### 2.4. Electrophysiological Responses in Adults

To measure antennal responses to *P. guineense* extract at concentrations of 0.3%, 1.5%, 3.0%, and 15% *v*/*v*, electroantennogram (EAG) experiments of 0–4-day-old virgin *S. frugiperda* males and females were performed with SFK and SFN populations. Each trial included two control treatments, with the pipettes either containing filter paper with 10 µL ethanol alone or completely empty. Additionally, we assessed responses of *S. frugiperda* male and female to the multicomponent blends (MCBs) of *S. frugiperda* sex pheromone containing five identified sex compounds as a positive control. The 5-component synthetic MCB, namely (Z)-9-tetradecenyl acetate (Z9-14:OAc), Z9-12:OAc, Z11-16:OAc, Z7-12:OAc, and E7-12:OAc, purchased from Pherobank (WijkbijDuurstede, The Netherlands) with >98% purity, was diluted in hexane to make a concentration of 10 ng/µL [35]. Sample sizes varied slightly by sex and population. For SFK: females (*n* = 6 per concentration and empty control; MCB: *n* = 2), males (*n* = 11 for all treatments). For SFN: females (*n* = 7; MCB: *n* = 3), males (*n* = 7 for all treatments). Each antenna was exposed to only one concentration of the extract to prevent sensory adaptation. However, due to the low number of replicates, MCB controls were excluded from statistical analysis but retained in the figures to provide a visual baseline for comparison with the extract concentrations and other controls.

The procedures described in Akinbuluma et al. [48] were slightly modified and used for the EAG setup. Specifically, in this study, we used crude plant extract dissolved in ethanol as stimuli; 10 μL of each extract concentration was applied to filter paper strips in Pasteur pipettes; and a broader range of concentrations (0.3–15% *v*/*v*) was tested independently on antennae from two geographically distinct *S. frugiperda* populations (Kenya and Nigeria). Live male or female insects were individually placed in a plastic pipette tip, and one antenna was immobilized with a small strip of parafilm pressing the antennal base against the head. Silver wires inserted in glass microelectrodes (GC150TF-10; Warner Instruments, Hamden, CT, USA) with insect Ringer’s solution were used to make electrical contact [49,50]. The reference electrode made contact with the cut antennal tip, while the recording electrode was inserted at the base of the antenna. The tip of the stimulus pipette was positioned approximately 1 cm from the midpoint of the insect antenna, aligned within the main airstream to ensure consistent stimulus delivery. The amplitude of the EAG was measured using an IDAC-4 amplifier (Syntech, Hilversum, The Netherlands) equipped with a high impedance (>10^9^ Ohm) head stage and recorded with EAD/2014 software (Syntech, Kirchzarten, Germany).

10 µL of each concentration in ethanol were pipetted onto a small piece of filter paper strip, 5 cm × 0.5 cm [51], and placed in a fume hood for 1 min to allow for evaporation of the solvent. The filter paper was later placed into a glass Pasteur pipette. A stimulus controller CS-55 V2 (Syntech, Hilversum, The Netherlands) produced a flow of 1 L/min charcoal-filtered air over the antennae. Antennae were stimulated with 0.5 s odor puffs, using a randomized sequence for each recording. The control stimuli were presented in between runs of the different concentrations. The inter-stimulus interval was 60 s. Signals were recorded with Syntech software (Autospike Version 3.9).

### 2.5. Mating and Oviposition Behavior in Presence of Extract

To determine whether *P. guineense* extract influenced mating in adult *S. frugiperda*, single-pair matings of SFK and SFN populations were set up. Virgin adults (1–4 days old) were set up in clear plastic cups (17 oz.) containing a small sauce cup (4 cm × 3 cm; 40 mL) filled with cotton wool soaked in a 10% sugar solution. Although individual mating competence was not pre-tested, all adults were reared under standard laboratory conditions and visually confirmed to be active and healthy prior to the experiment. Matings were set up in a climate chamber (26 °C, 70% RH, L:D 14:10) at two hours before scotophase, the period of darkness in a 24-h light-dark cycle; see Yuan et al. [52]. In each mating cup, 10 μL of either 0.3%, 1.5%, 3.0%, or 15% *P. guineense* concentrations were added to a small strip of filter paper (5 cm × 0.5 cm), which was covered with muslin cloth, for slow evaporation and to ensure a gradual release of volatiles. The controls included two mating cups that received 10 μL of ethanol only, which we refer to as 0% (*v*/*v*) *P. guineense* concentration, and two mating cups that did not receive any paper strip (empty control).

Each treatment was replicated four times with one mating pair per replicate, resulting in a total of 48 mating pairs (4 mating pairs × 6 treatments × 2 populations) and arranged in a completely randomized design. Couples were observed throughout the ten hours of scotophase, facilitated by a dim red light to avoid disrupting normal nocturnal behavior. Observations were made at 30 min intervals for three consecutive nights after setup. Furthermore, oviposition by *S. frugiperda* females was recorded daily during the observation period by counting the number of egg masses in each cup.

### 2.6. Female Behavioral Responses to Extracts

To determine behavioral responses of adult *S. frugiperda* females to *P. guineense* extract, we conducted a wind tunnel assay, using a procedure modified from Sobhy et al. [53]. 10 µL of the extract at a concentration of 1.5% (*v*/*v*) or ethanol (control) was applied to a piece of filter paper, placed inside lure holders, and hung 40 cm apart on a retort stand. The retort stand was positioned at the upwind end of a wind tunnel (200 cm long  ×  100 cm wide  ×  100 cm high plexiglass flight section). The odor sources were positioned 50 cm from the upwind wall of the tunnel. The wind tunnel was lit from above by red LED strips, and the experiment was conducted under controlled environmental conditions (27 °C, 22% RH) and a wind speed of 0.24 m/s (using an anemometer). Air entering the tunnel was filtered through activated charcoal and humidified to ensure a clean, laminar flow. Odor delivery was continuous throughout a 12-min trial.

Adult female moths (2-day-old) were allowed to acclimate to the tunnel room for 1 h prior to testing. A total of 20 females from each *S. frugiperda* population (SFK and SFN) were tested individually. The moths were individually introduced through a side panel at the downwind end of the wind tunnel (130 cm from the odor release point) from an elevated release point that matched the height of the odor plume on a retort stand placed inside the tunnel. For each moth taking flight and first landing within a 12 min period, the distance from the source (control or test) was recorded in five distance classes: 0–5 cm, 5–10 cm, 10–15 cm, 15–20 cm, and 50 cm (for any choice farther away than 20 cm). Odor sources were swapped after 4 months of testing, and the bioassay was conducted between 15:00 and 23:00.

### 2.7. Statistical Analyses

All analyses were performed in R (version 4.3.2) [54]. Larval mortality was analyzed using a generalized linear mixed model (GLMM), whereby insect mortality was set as the response variable, while population, concentration, day, and their interactions were set as fixed variables. Replicate was added as a random effect. LC_50_ values were calculated for each day with the dose–response model (DRM) from the dose–response curve (DRC) package (version 3.0-1) [55], and the differences were compared using the compParm and confint functions from the same package. Differences in the number of pupae and pupal weight between control and treatments were analyzed with GLMs. For pupation (yes or no), a binomial distribution was used and a Gaussian distribution for pupal weight. In both models, concentration and population were the explanatory variables. Post hoc testing was performed with estimated marginal means [56] and Tukey correction for multiple comparisons.

The electrophysiology data was analyzed with a linear mixed model (LME). The response variable was amplitude, which was square root transformed to meet the assumptions of homoscedasticity and normality. The fixed variables were population, exposure, sex, and their interactions, and replicate was included as a random effect. To analyze the impact on mating probability, we used a GLMM with a binomial distribution. Population and exposure were fixed variables, and replicate set was a random effect.

In the case of length of mating time, we used LME and set mating length as the response variable, while population, treatment, and time period were the fixed variables, and again, replicate was the random effect. However, the variance and standard deviation for the fixed effect in the model were both zero. Therefore, the random effect was removed, and a linear model was used instead. The response variable and fixed variables remained the same. To assess the impacts of extracts on oviposition, a GLMM with a Poisson distribution was used. Cumulative oviposition was used as the response variable, and population, exposure, and their interactions as the fixed variables. Replicate was included as a random effect.

For the behavioral responses of females in the wind tunnel experiment, the final dataset contained 32 insects from 2 populations (SFK and SFN), as 8 females did not respond within the time frame of 12 min, collected over two measuring days (day 1 or day 2). To determine if population, day, time in scotophase, and distance from source influenced female choices, a binomial GLM was constructed, with the first landing side as the response. We tested the zero hypothesis of equal probability of choice for the treatment and control side with the binomial test and the effect of distance to source on side.

In all cases, a model simplification procedure was followed. The full model was fitted, after which the least significant term (from the three-way interaction downwards) was removed if the removal resulted in an insignificant increase in deviance. The significance of simplified models was assessed by performing a likelihood ratio test. The model simplification process was repeated until the model contained only significant terms (level *p* < 0.05). To check for overdispersion and nonuniformity of the residuals in the fitted models, we used the Residual Diagnostics for HierARchical Models (DHARMa) package (version 0.4.6) [57]. Our final models had no significant overdispersion or nonuniformity of the residuals.

## 3. Results

### 3.1. Larval Responses to Extract

**Figure 1:**
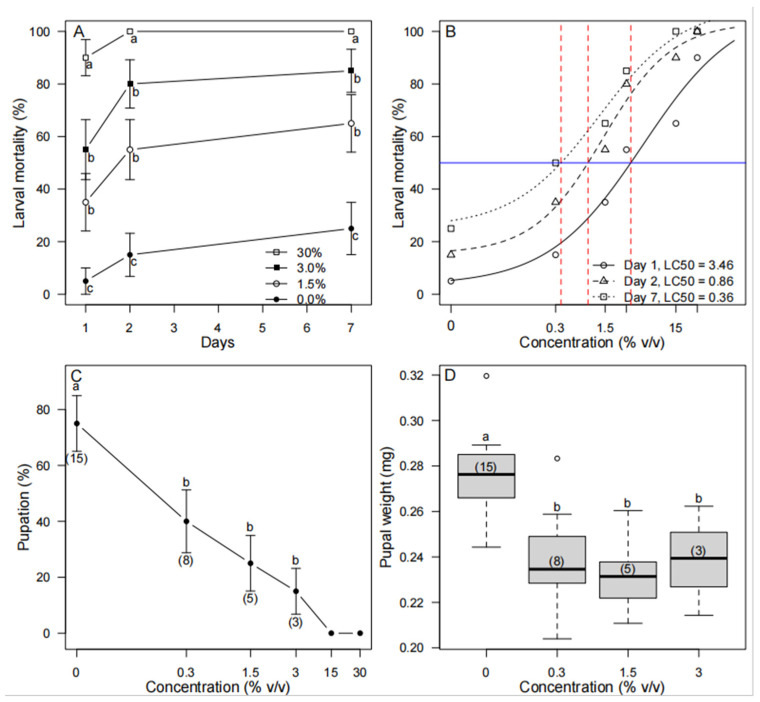
Effect of contact toxicity of *Piper guineense* on *Spodoptera frugiperda* larvae and pupae. (**A**). Effects of different concentrations of *P. guineense* extract on *S. frugiperda* larvae mortality after one, two, and seven days of exposure (0%—solid circle; 1.5%—open circle; 3.0%—solid square; 30%—open square). For visual clarity, concentrations of 0.3% *v*/*v* and 15% *v*/*v* were excluded from the graph because the concentrations were not significantly different from the control (0%) or the highest concentration (30%), respectively. Comparisons were made between concentrations for each day (*n* = 20). (**B**). Log-linear concentration response curves for the three days of observation (Day 1—open circle and solid line; Day 2—open triangle and dashed line; Day 3—open square and dotted line). The LC_50_ is indicated by the red line. (**C**). Percentage pupation (i.e., number of surviving pupae) of *S. frugiperda* larvae at different concentrations of the extract (number of surviving insects in each concentration is presented in parentheses under the error bars). (**D**). Weights of *S. frugiperda* pupae after exposure of larvae to concentrations of *P. guineense* extract. The number of surviving insects in each concentration is given in parentheses within each box. In (**A**,**C**,**D**), different letters indicate significant differences across treatments (*p* < 0.05).

Topical application of *P. guinense* to *S. frugiperda* larvae showed a strong concentration-response for all the extract concentrations across days of the trials (Figure 1). We found a significant effect of the extract concentrations (df = 338, *p* = 0.000) and day (df = 338, *p* = 0.001), but no significant effect of population or any interaction effect. The highest mortality (100%) of *S. frugiperda* larvae was observed with 30% (*v*/*v*) of *P. guinense* extract. Except, with a 0.3% (*v*/*v*) concentration of *P. guineense*, mortality of *S. frugiperda* larvae in other treatments was significantly different (*p* < 0.05) from the mortality in the control. The LC_50_ values of *P. guineense* (ranging between 0.86% and 3.46%) were not significantly different between days 1, 2, and 7 (Table S1a,b). Percentage pupation was significantly higher in the ethanol control than in the *P. guineense* treatments, and none of the larvae made it to pupation at concentrations ≥15% (*v*/*v*). Also, the pupae in the control treatment weighed significantly more than pupae from the *P. guineense* extract-treated larvae (*p* < 0.05).

### 3.2. Electrophysiology

**Figure 2:**
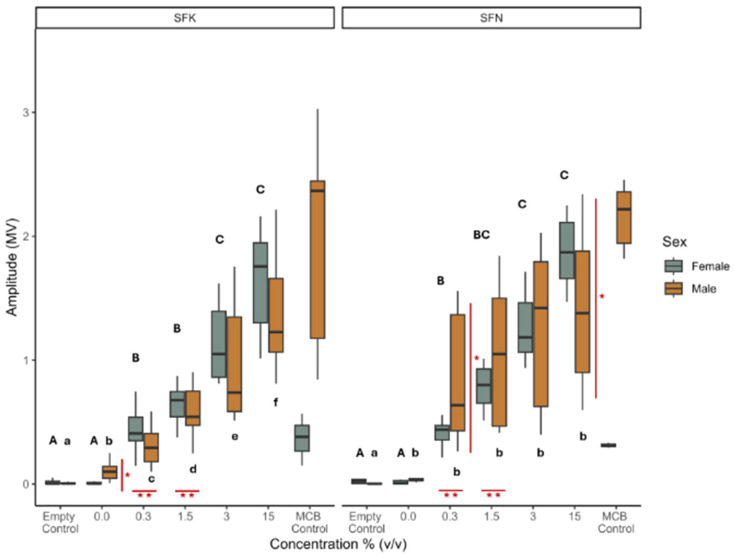
Electrophysiological responses of *Spodoptera frugiperda* females and males from Kenya (SFK) and Nigeria (SFN) to *Piper guineense* extracts. Box and whisker plots of electroantennography (EAG) responses (amplitude in millivolt (mV)) of the SFK and SFN populations and both sexes. The left panel represents the SFK population, and the right panel the SFN population, with light grey indicating responses of females and orange the male responses. Extract concentrations of *P. guineense* are in percentage volume to volume (% (*v*/*v*)). The empty control represents a negative control. The synthetic multicomponent blend (MCB), containing five female sex pheromone compounds of *S. frugiperda*, was included as a visual comparison only and not included in the analysis due to low sample size. Differences in capital letters denote significant differences between exposure to treatments of varying extract concentrations for females within a population; differences in small letters denote significant differences between treatments for males within a population. The red vertical line with a single star, to the right of a box plot, indicates significant differences between males and females at the given exposure treatment. The red horizontal line with two stars, below box plots, indicates significant differences between populations for a given exposure treatment. The significance level was set at *p* < 0.05. Sample sizes varied between populations and sexes. SFK—Female: empty control and all concentrations (0% (*v*/*v*)–15%(*v*/*v*)), *n* = 6, MCB control *n* = 2; Male: empty control, all concentrations, and MCB control *n* = 11. SFN—Female: empty control and all concentrations, *n* = 7, MCB control *n* = 3; Male: empty control, all concentrations, and MCB control *n* = 7.

In assessing the electrophysiological responses, the multicomponent (MCB) control only had a few samples for females of both populations (*n* = 2 for SFK and *n* = 3 for SFN). Given this low sample size, responses to MCB were excluded from the analysis but included in the figure for visual comparison with other concentrations used. We found that the response of *S. frugiperda* from both populations and sexes differed when exposed to *P. guineense* extract concentrations; an increase in extract concentration of *P. guineense* led to an increase in EAG response in both populations and both sexes. We found a significant interaction effect between population, sex, and the extract concentration (LME Population: Exposure: Sex; estimate = 0.51 ± 0.17 se, df = 163.78, t-value = 2.98, *p* = 0.003) (Figure 2).

Males of the two populations differed in their response to an exposure of 0.3% (*v*/*v*) (pairs contrast: estimate = 0.34 ± 0.08, df = 169, t-ratio = 4.27, *p* < 0.0001) and 1.5% (*v*/*v*) concentration (pairs contrast: estimate = 0.22 ± 0.08, df = 169, t-ratio = 2.74, *p* = 0.007) (Figure 2, horizontal red lines and two stars underneath the bars). When comparing the response between sexes within each population, in the SFK population, females and, males differed in their EAG response at the 0% (*v*/*v*) extract concentration (pairs contrast: estimate = 0.22 ± 0.08, df = 169, t-ratio = 2.80, *p* = 0.006), with males having a higher response than females. In the SFN population, the sexes differed in their EAG response at the 0.3% (*v*/*v*) extract concentration (pairs contrast: estimate = 0.26 ± 0.09, df = 165, t-ratio = 2.77, *p* = 0.006), with males having a higher response than females, and the 15% (*v*/*v*) extract concentration (pairs contrast: estimate = −0.20 ± 0.09, df = 165, t-ratio = −2.20, *p* = 0.029), with females having a higher response than males (Figure 2, vertical red lines and single stars).

### 3.3. Effects of Extracts on Mating and Oviposition

**Figure 3:**
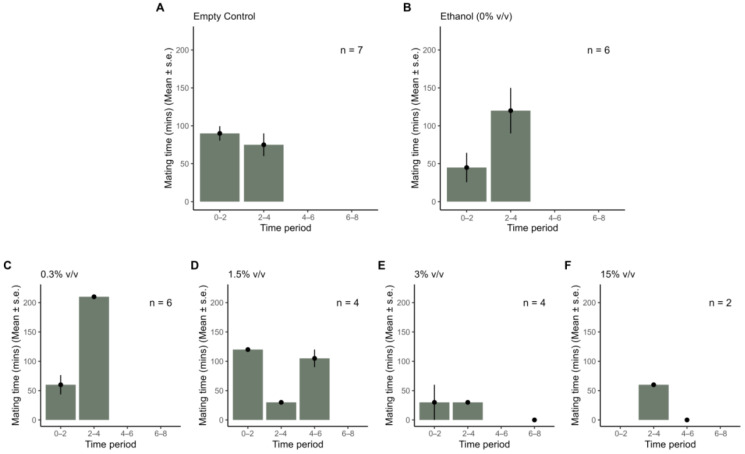
Duration of mating of *Spodoptera frugiperda* as influenced by exposure to *Piper guineense* extract within ten hours. (**A**) Empty control containing paper strip only; (**B**) paper strip exposed to ethanol (equivalent to 0% (*v*/*v*)); (**C**–**F**) paper strips treated with *P. guineense* extract at concentrations of 0.3%, 1.5%, 3%, and 15% (*v*/*v*). There was no significant difference between treatments or time periods.

**Figure 4:**
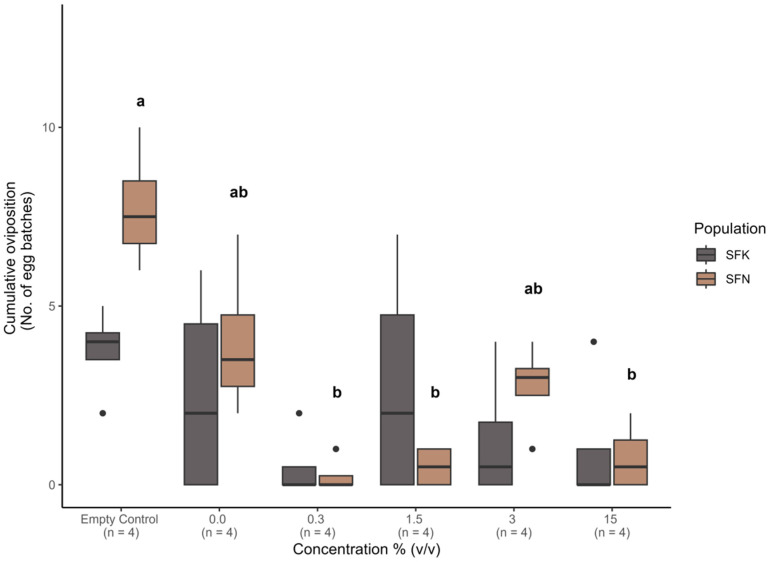
Effect of *Piper guineense* extract concentrations on *Spodoptera frugiperda* oviposition. Box and whisker plots of the number of egg batches of females exposed to *P. guineense* extract at concentrations of 0.3%, 1.5%, 3%, and 15% *v*/*v*. Dark grey boxes represent females from the Kenya population (SFK), and dark cream boxes represent females from the Nigeria population (SFN). Different small letters within the graph represent significant differences (*p* < 0.05) between treatments in the SFN population. The SFK population showed no significant difference between exposure treatments.

Mating probability was similar in all treatments (Figure S1). Model results indicated a significant declining trend in mating probability when comparing an exposure concentration of 15% (*v*/*v*) with the empty control, where an exposure concentration of 15% (*v*/*v*) had a significantly lower probability of mating than the empty control (estimate = 3.21 ± 1.40 se, z-value = −2.29, *p* = 0.02). However, after Tukey correction for multiple comparisons, this difference was no longer significant (estimate = 3.21 ± 1.40 se, z-ratio = 2.29, *p* = 0.199). Mating time (duration) varied across exposure concentrations and time periods (Figure 3) but was not significantly affected by population (ANOVA: df = 1, F = 0.002, *p* = 0.968), exposure concentration (ANOVA: df = 5, F = 1.42, *p* = 0.26), or time period (ANOVA: df = 3, F = 1.20, *p* = 0.33). There was a significant interaction between population and extract concentration on oviposition (GLMM: estimate = −2.43 ± 0.83 se, z-value = −2.93, *p* = 0.003). The SFK population did not show any difference in oviposition with increasing extract concentration (Figure 4, Table S2). However, in the SFN population we found reduced oviposition at the 0.3% (estimate = 3.43 ± 1.02 se, z-ratio = 3.38, *p* = 0.03), 1.5% (estimate = 2.74 ± 0.73 se, z-ratio = 3.76, *p* = 0.009), and 15% (estimate = 2.34 ± 0.60 se, z-ratio = 3.87, *p* = 0.006) extract concentrations when compared to the empty control (Figure 4, Table S2).

### 3.4. Behavioral Response of Females to Extracts

**Figure 5:**
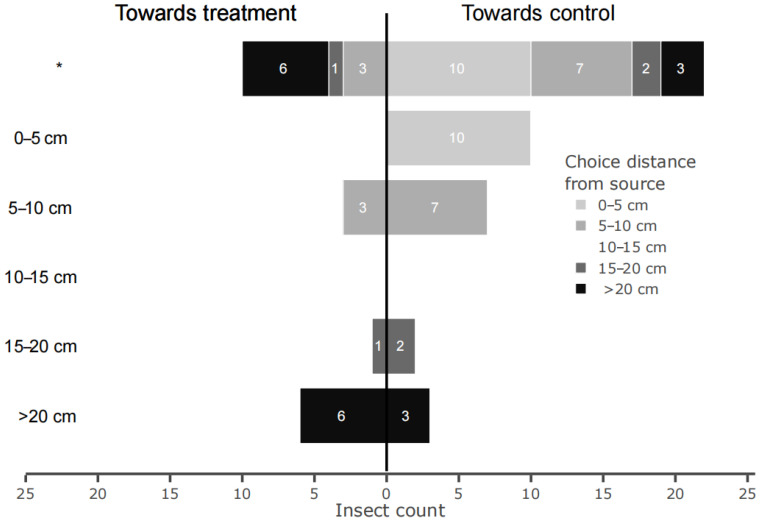
Repellent effect of *Piper guineense* extract on adult female *Spodoptera frugiperda*. First landing responses (towards treatment or control) of adult *S. frugiperda* females (*n* = 32) by the distance to the source measured within a time frame of 12 min are represented by the first line of bars. Landing responses are split by the distances of the choices (indicated by the direction of the bars) for treatment or control by each insect. Numbers inside the bars represent the number of responses. The star represents significant difference between the number of *S. frugiperda* females that chose the *P. guineense* odor source over the control odor source at the measured distances.

In the wind tunnel experiment, we found no difference in response between SFK and SFN females (df = 28, *p* = 0.127) or between the time of measurements within the scotophase (df = 28, *p* = 0.923). However, a significantly lower number of *S. frugiperda* females chose the *P. guineense* odor source over the control odor source at the measured distances (*p* = 0.019) (Figure 5). Females that landed closer towards the odor sources (0–5 or 5–10 cm) landed more often towards the control odor than towards the plant odor source (GLM, *p* = 0.008, df = 30), while females that landed at a further distance from the source were less repelled.

## 4. Discussion

Many tropical plants produce secondary compounds to protect themselves against herbivores and pathogens, and some have been used for pest management [16,17,58,59]. Apart from causing death, botanical insecticides can disrupt the biological activity of insect pests, including suppression of feeding activity, reduction of pupal weights, prolonging developmental time, and influencing mating and oviposition, and their odors can have repellent effects [60,61,62,63].

In this study, we tested the effect of *P. guineense* extract on different life stages of *S. frugiperda* because this is a promising pesticidal plant species to manage this pest. The response of *S. frugiperda* larvae to the topical applications of *P. guineense* extracts showed a positive relationship between concentration, time of exposure, and mortality. This agrees with the results of Lina et al., who reported that an increase in the larval mortality of *S. frugiperda* was proportional to the increase in the testing concentration [33]. As *Piper guineense* extract caused up to 100% mortality at 30% (*v*/*v*) of the extract after 2 days of exposure, the high larvicidal effect of this plant is obvious. In general, botanical pesticides cause 80% mortality under laboratory conditions [64]. In Ethiopia, *Nicotiana tabacum* caused only 50% mortality after 72 h exposure to third instar *S. frugiperda* larvae [21], a value that is considerably lower than the mortality observed in our bioassay. Neem extracts [20,65] and extracts from *Eucalyptus urograndis* [66] were very effective in causing mortality of *S. frugiperda.*

It is likely that the mortality caused by the extract of *P. guineense* is related to the secondary metabolites present in the extract. Earlier phytochemical and chromatographic analyses revealed the presence of a range of secondary metabolites in *P. guineense* solvent fractions, including phenolic compounds, alkaloids, and terpenoids [35,67]. For example, terpenoids and alkaloids often interact with specific targets, such as receptors or certain enzymes, thereby interfering with particular cellular pathways in insects [68]. Alkaloids are known to act on various metabolic systems in animals, including insects. Some can disrupt enzyme functions and alter different physiological processes, while others bind to nucleic acids, thereby inhibiting DNA synthesis and repair, and others have strong effects on the nervous systems [68,69]. Piperamides such as piperine, a major compound in *P. guineense* [39,70,71], may exert neurotoxic effects by disrupting insect octopaminergic signaling, leading to hyperexcitation, paralysis, and death. Piperamides also inhibit cytochrome P-450-dependent polysubstrate monooxygenase (PSMO) in insects, preventing the detoxification of toxicants and enhancing the effect of the toxic insecticides [72,73].

The fact that we found high levels of mortality already within one day of exposure to the plant extract (not significantly different from other days of observation) suggests a rapid toxic action on the insects, which is in line with other reports [29,33,61,74]. The plant extract also resulted in significantly lower percentages of pupation and lighter pupae in the insects that survived, which can add to its effectiveness. Possibly, reduced pupal weights and pupation rates result from interference with hormonal regulation of growth and development of the insect [75,76]. When testing the potential of three botanicals, including *Piper aduncum* on *S. frugiperda*, *P. aduncum* did not only prolong larval development but also caused lower pupal weight than those in the control [61]. These results support the possibility that *P. guineense* extract may disrupt the physiological processes of *S. frugiperda*.

Overall, an increase in extract concentration of *P. guineense* led to an increase in EAG response in both Kenyan and Nigerian populations of *S. frugiperda*, revealing that antennal responses to the extract were concentration-dependent. That the adult male and female *S. frugiperda* responded differently to the highest concentration of *P. guineense* signifies a sex difference in the electrophysiological response of the insect. This may be attributed to sexual dimorphism in antennal morphology and olfactory receptor expression [77,78]. To our knowledge, this is the first report on the EAG responses of male and female *S. frugiperda* to *P. guineense* extract. Further future tests may be needed to identify and profile olfactory receptors that could help clarify the mechanisms underlying sex-specific responses of *S. frugiperda* to plant compounds.

Although we included the MCB of synthetic sex pheromone blends for visual comparison only, we observed that, relative to the extract concentrations, the MCB produced high EAGs on male *S. frugiperda* from both SFK and SFN populations. Several authors have reported that sex pheromone compounds released by female *S. frugiperda* elicited responses from conspecific males and have been used to capture the latter [79,80,81,82]. However, only little EAG responses were observed with the female populations, agreeing with earlier evidence that females of certain Lepidoptera species [83,84], including *S. frugiperda* [85,86], can autodetect their sex pheromone. More studies involving this autodection of *S. frugiperda* to their sex pheromones are needed.

We found no significant differences in the mating duration between mating couples treated with *P. guineense* extract and untreated controls, suggesting that the extract did not disrupt mating at the concentrations tested. This finding is in contrast to Sammani et al., who reported a greater percentage of successful mating in *Cadra cautella* (Lepidoptera: Pyralidae) when exposed to camphor oil, citronella oil, and neem oil [87]. Therefore, plant extracts may either boost mating success among moths or have no effect on mating.

Furthermore, we found that oviposition of the *S. frugiperda* Nigerian population was influenced by *P. guineense* extract, as fewer egg batches were laid at higher concentrations. Previous studies also found that *Piper hispidinervum* C. affected spermatogenesis and egg laying in *S. frugiperda* [22,88]. However, we did not observe any difference in egg batches in the Kenya population as a result of treatment with *P. guineense* extract. In the Indian meal moth *Plodia interpunctella* (Hübner), botanical extracts from olive, sunflower, and corn did not reduce oviposition but enhanced it on several food hosts [89], suggesting that different populations may behave differently to different plant extracts. More research is needed to test the effect of extracts of different plants on different populations of *S. frugiperda.*

In the wind tunnel bioassay, we found that *S. frugiperda* females landed more in the vicinity of the ethanol control than the *P. guineense* odor source. Previously, a repellent effect of *Piper* extracts was also found on *S. frugiperda* larvae [31,34]. In addition, *Piper* extract repelled coleopterous insect pests in storage, such as the banana weevil, *Cosmopolites sordidus* (Germar) [90], and the maize weevil, *Sitophilus zeamais* [35]. However, our observation that at further distances from the odor source the repellent effect of *P. guineense* disappeared suggests that repellence only works at short distances. Although a single concentration was used in the flight assays, future flight-behavior assays should test multiple extract concentrations to establish a full dose–response profile and correlate antennal sensitivity with behavioral outcomes.

Many activities of extracts from *Piper* species, including their repellent effect, are related to the secondary metabolites in the extract, including alkaloids, triterpenoids, tannins, coumarins, and terpene lactones [91,92]. Among the several secondary metabolites in plants, alkaloids have been reported to largely affect the nervous system, regulate growth, and act as a repellent against *S. frugiperda* larvae [31,91,93]. Therefore, the repellent effect of *P. guineense* in this study may largely be due to the numerous alkaloids [39].

## 5. Conclusions

Our study demonstrated that *Piper guineense* extract was toxic to *Spodoptera frugiperda* in a concentration-dependent way. Specifically, the extract showed larvicidal activity against *S. frugiperda*, produced high electrophysiological responses in both adult males and females, and was repellent to females at close distance. To advance the practical application of *P. guineense*, future research should investigate the exact mode of action(s) of the extract, assess its efficacy under field conditions, and incorporate comparisons with established commercial biopesticides such as neem-based products or *Bacillus thuringiensis* in the management of *S*. *frugiperda*.

## Data Availability

The authors confirm that data supporting the findings of this study are available from the corresponding author on request.

## Supplementary Materials

The following supporting information can be downloaded at: https://www.mdpi.com/article/10.3390/insects16090908/s1, Table S1a: Statistical comparison of LC_50_ values of *Piper guineense* extract on *S. frugiperda* larvae tested by topical application between days, Table S1b: LC_50_ values of *Piper guineense* extract across days against *S. frugiperda* larvae tested by topical application, Figure S1: Probability of mating of *S. frugiperda* exposed to extract concentrations of *P. guineense* extract, Table S2: Contrast effects of extract concentration and population on oviposition, Figure S2: EAG responses of *Spodoptera frugiperda* males and female 56 for combined SFK and SFK populations to extract concentration of *Piper guineense*.

## Abbreviations

The following abbreviations are used in this manuscript.

EAG	Electroantennogram
LC_50_	Lethal concentration
SFK	*Spodoptera frugiperda* from Kenya
SFN	*Spodoptera frugiperda* from Nigeria
ICIPE	International Centre of Insect Physiology and Ecology
IITA	International Institute of Tropical Agriculture
UvA	University of Amsterdam
DHARMa	Residual Diagnostics for HierARchical Models
MCB	Multicomponent blend
RH	Relative Humidity
L:D	Light:Dark
DRM	Dose–response model
DRC	Dose–response curve
LME	Linear mixed model
GLMM	Generalized linear mixed model
mV	Millivolt
μL	Microliter
mL	Milliliter
