# Supplementary material for: Contact Toxicity, Electrophysiology, Anti-Mating, and Repellent Effects of Piper guineense Against Spodoptera frugiperda (Lepidoptera: Noctuidae)
Source: Insects. 2025 Sep 1;16(9):908. doi: 10.3390/insects16090908 (PMC12470923; doi:10.3390/insects16090908)
Supplement: Supplementary file 1 [file insects-16-00908-s001.zip › insects-3788368-supplementary.pdf]

Contact toxicity, Electrophysiology, Anti-mating and Repellent effects of *Piper guineense* against *Spodoptera frugiperda* (Lepidoptera: Noctuidae)

Mobolade D. Akinbuluma[1,2]*, Jacques A. Deere[1], Peter Roessingh[1] and Astrid T. Groot[1]

[1] Department of Evolutionary and Population Biology, Institute for Biodiversity and Ecosystem Dynamics, Amsterdam, Netherlands; m.d.akinbuluma@uva.nl (M.D.A); j.a.deere@uva.nl (J.A.D); p.roessingh@uva.nl (P.R); a.t.groot@uva.nl (A.T.G)
[2] Department of Crop Protection and Environmental Biology, University of Ibadan, Ibadan, Nigeria; md.akinbuluma@ui.edu.ng (M.D.A)
* Correspondence: m.d.akinbuluma@uva.nl; Tel.: +31-6-86 27 9835

Supplementary Information

Table S1a. Statistical comparison of LC_{50} values of *Piper guineense* extract on *Spodoptera frugiperda* larvae tested by topical application between days

Comparison across days	Estimate	Std. Error	t-value	*p*-value
1-2	3.69989	9.8652	0.3750	0.7109
1-7	3.88432	9.8759	0.3933	0.6976
2-7	0.18443	0.9821	0.1878	0.8526

LC_{50} values were not significantly different ($p > 0.05$) across pairs of days.

Table S1b. LC_{50} values of *Piper guineense* extract across days against *Spodoptera frugiperda* larvae tested by topical application

Time (Days)	LC_{50}	95% CI
1	3.46	-15.22-25.43
2	0.86	0.14- 2.67
7	0.36	-0.36- 2.80

LC_{50} values were not significantly different ($p > 0.05$) due to overlapping respective 95% CI. LC_{50} = median lethal concentration; CI = confidence interval.

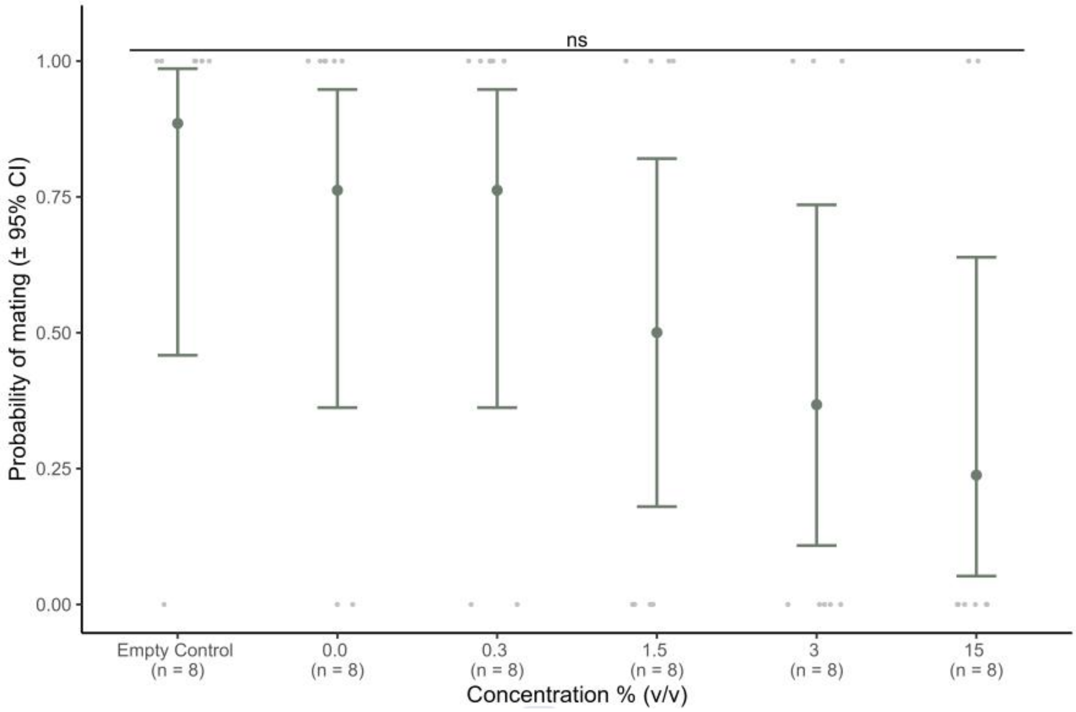

Figure S1: Probability of mating of *Spodoptera frugiperda* exposed to extract concentrations of *Piper guineense* extract. ns = mating probability not significant (p > 0.05). Grey dots indicate raw data.

Table S2. Contrast effects of extract concentration and population on oviposition. Contrasts are Tukey *p*-value adjustments after a significant interaction between population and extract concentration was found (GLMM: estimate = -2.43±0.83se, z-value. = -2.93, p = 0.003). Values are given on a log scale as the initial model was fitted with a Poisson distribution. SFK = *Spodoptera frugiperda* Kenya; SFN = *Spodoptera frugiperda* Nigeria

Contrast	estimate	s.e	z-ratio	p-value
SFK population				
Empty Control - 0.0%	0.41	0.41	0.99	1.00
Empty Control - 0.3%	2.01	0.75	2.68	0.24
Empty Control - 1.5%	0.31	0.40	0.78	1.00
Empty Control - 3%	1.10	0.52	2.13	0.60
Empty Control - 15%	1.32	0.53	2.35	0.44
0.0% - 0.3%	1.61	0.77	2.08	0.64
0.0% - 1.5%	-0.09	0.44	-0.22	1.00
0.0% - 3%	0.69	0.55	1.27	0.98
0.0% - 15%	0.92	0.59	1.55	0.93
0.3% -1.5%	-1.70	0.77	-2.22	0.53
0.3% - 3%	-0.92	0.84	-1.10	0.99
0.3% -15%	-0.69	0.87	-0.80	1.00
1.5% - 3%	0.79	0.54	1.46	0.95
1.5% - 15%	1.01	0.58	1.74	0.85

3% - 15%	0.22	0.67	0.33	1.00
SFN population				
Empty Control - 0.0%	0.66	0.31	2.15	0.59
Empty Control - 0.3%	3.43	1.02	3.38	**0.03**
Empty Control - 1.5%	2.74	0.73	3.76	**0.009**
Empty Control - 3%	1.04	0.35	2.96	0.12
Empty Control - 15%	2.34	0.60	3.87	**0.006**
0.0% - 0.3%	2.77	1.03	2.69	0.23
0.0% - 1.5%	2.08	0.75	2.78	0.19
0.0% - 3%	0.37	0.39	0.96	0.99
0.0% - 15%	1.67	0.63	2.66	0.24
0.3% -1.5%	-0.69	1.22	-0.57	1.00
0.3% - 3%	-2.40	1.04	-2.30	0.48
0.3% -15%	-1.10	1.15	-0.95	1.00
1.5% - 3%	-1.70	0.77	-2.22	0.53
1.5% - 15%	-0.41	0.91	-0.45	1.00
3% - 15%	1.30	0.65	2.00	0.70

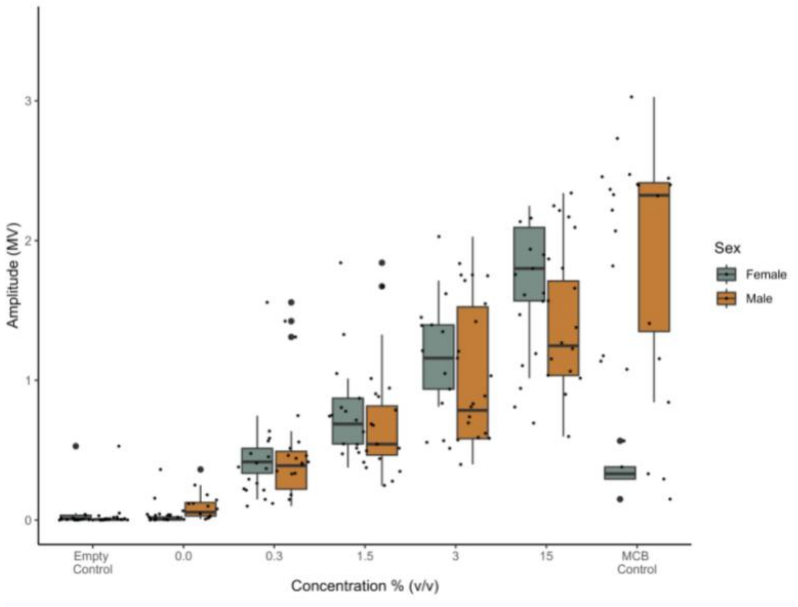

Figure S2: EAG responses of *Spodoptera frugiperda* males and female for combined SFK and SFK populations to extract concentration of *Piper guineense*. Box and whisker plots indicate amplitude response (in millivolts (mV)) of females (light grey) and males (orange). Extract concentrations of *P. guineense* are in percentage volume to volume (% (v/v)). The empty control represents negative control and the MCB (multiple component blends of the five pheromone compounds from a *S. frugiperda* female) represents a positive control. Sample sizes per sex: Female – Empty control and all concentrations (0.0% (v/v) - 15% (v/v)) = 13, MCB control = 5; Male – Empty control, all concentrations and MCB control = 18). Black dots indicate raw data.
